# Alternative Splicing of *PheNAC23* from Moso Bamboo Impacts Flowering Regulation and Drought Tolerance in Transgenic *Arabidopsis*

**DOI:** 10.3390/plants13233452

**Published:** 2024-12-09

**Authors:** Lihua Xie, Xiangyu Li, Pengqiang Yao, Zhanchao Cheng, Miaomiao Cai, Chunyang Liu, Zhe Wang, Jian Gao

**Affiliations:** 1 Henan Key Laboratory of Germplasm Innovation and Utilization of Eco-Economic Woody Plant, Pingdingshan University, Pingdingshan 467000, China; xielihua0227@163.com (L.X.); yaopengqiang1988@163.com (P.Y.); chyliu2013@163.com (C.L.); 27101@pdsu.edu.cn (Z.W.); 2 Key Laboratory of National Forestry and Grassland Administration/Beijing for Bamboo & Rattan Science and Technology, International Center for Bamboo and Rattan, Beijing 100102, China; leerduo727@163.com (X.L.); czc@icbr.ac.cn (Z.C.); cmm@icbr.ac.cn (M.C.); 3 State Key Laboratory of Subtropical Silviculture/Bamboo Industry Institute, Zhejiang A&F University, Hangzhou 310000, China

**Keywords:** drought stress, moso bamboo, NAC transcription factor, alternative splicing

## Abstract

NAC (NAM, ATAF, and CUC) transcription factors are essential in regulating plant stress response and senescence, with their functions being modulated by alternative splicing. The molecular mechanisms of stress-induced premature flowering and drought tolerance in *Phyllostachys edulis* (moso bamboo) are not yet fully understood. In this study, a novel NAC variant derived from *PheNAC23*, named *PheNAC23^ES^*, was isolated. PheNAC23^ES^ exhibited distinct expression patterns compared to PheNAC23 during leaf senescence and drought stress response. Overexpression of *PheNAC23* promoted flowering and reduced its tolerance to drought stress in *Arabidopsis thaliana* (*A. thaliana*). However, overexpression of *PheNAC23^ES^* exhibited the opposite functions. PheNAC23 was localized in the nucleus and had transactivation activity, while PheNAC23^ES^ had a similar localization to the control green fluorescent protein and no transactivation activity. Further functional analysis revealed that PheNAC23^ES^ could interact with PheNAC23, suggesting that PheNAC23^ES^ might serve as a small interfering peptide that affects the function of PheNAC23 by binding to it.

## 1. Introduction

Moso bamboo is an important source of forest products, regenerates annually as clones and occupies approximately 72.9% of the total area of bamboo forests [1]. Moso bamboo has been used as an industrial raw material and food for decades and is beneficial for both its ecological and economic values. However, the detrimental effects of drought stress, exacerbated by climate change, have significantly impacted the development and distribution of moso bamboo. Drought is the most devastating stress, adversely affecting plant growth and productivity, particularly for moso bamboo [2]. In moso bamboo, drought stress is considered to be related to bamboo flowering [3], which is a signal of bamboo stand aging. However, the relationship between drought stress and bamboo stand aging is still unknown.

Transcription factors (TFs) are typical nucleoproteins containing a distinct type of DNA-binding domain and transcriptional regulation region. These transcription factor proteins activate or repress the transcription of target genes by binding to their promoter regions [4]. NAC, named NAM (No apical meristem domain), ATAF (Arabidopsis transcription activator factor), and CUC (Cups shaped cotyledon) protein, is a large TF family widely distributed in plants. The NAC proteins comprise a conserved NAM domain of DNA binding in the N-terminus and a variable transcriptional regulation region at the C-terminus [5]. The conserved NAM domain comprises approximately 160 amino acid residues. It is divided into five subdomains from A to E. The highly conserved subdomain A may be involved in dimer formation, while the highly conserved C and D subdomains participate in DNA binding. The relatively non-conserved B and E subdomains may be related to the diversity of gene functions in the NAC family [6,7]. The NAC domain-fold also modulates dimerization through Leu (Leucine)14–Thr (Threonine)23 and Glu (Glutamic acid)26–Tyr (Tyrosine)31 residues, which form a short antiparallel b-sheet at the dimer interface, stabilized by salt bridges formed by Arg (Arginine)19 and Glu (Glutamic acid)26, in subdomain A [8,9]. Multiple studies have shown that NAC TFs regulate plant growth and development and respond to environmental stresses [10]. For example, overexpression of salt-responsive *AtNTL8* delayed flowering and reduced the leaf growth of Arabidopsis by decreasing the expression of *FLOWERING LOCUS T* (FT) [11]. Interestingly, NACs regulate plant development by interacting with diverse stresses, including cold and drought. *AtLOV1* confers cold tolerance in Arabidopsis and controls flowering time within the photoperiod pathway [12]. In *Oryza sativa* (rice), overexpression of *ONAC11* (*OMTN4*), negatively related to drought stress, promotes leaf senescence and early flowering [13,14]. These studies suggest that some NACs can act as hubs linking stresses to plant flowering.

The discovery of alternative splicing (AS) is often regarded as a significant milestone in enhancing our comprehension of plant post-transcriptional mechanisms [15]. AS mechanisms modulate transcript types and levels by generating multiple transcripts from a single pre-mRNA, thereby enhancing proteome complexity [16]. Small proteins (peptides) produced through AS, which may possess dimerization domains but lack other functionalities, function as dominant negative regulators by competitively forming nonfunctional heterodimers with functional transcription factors [17]. Target genes regulated by alternative splicing include splicing regulators, TFs, protein kinases, and pathogen-resistance genes. These genes confer enhanced stress tolerance and cater to developmental needs by modulating the abundance of specific isoforms [18]. In *Populus trichocarpa* (*P. trichocarpa*), *PtrSND1-A2^IR^* is an intron-retained splice variant derived from *PtrSND1-A2* mRNA. This translocated PtrSND1-A2^IR^ lacks DNA-binding and transactivation abilities, and it can disrupt the function of full-size PtrSND1s by forming nonfunctional heterodimers with them. This process modulates the SND1 transcriptional network, rendering the PtrSND1s nonproductive [19]. In rice, trans-splicing between the genes *ONAC020* and *ONAC026* generates three additional forms of *ONAC020*. These alternative splice forms may function as competitors or interfering peptides within a network that regulates seed size and weight in different rice accessions [20]. The transcriptional isoforms PtATAF1.2b and PtATAF 1.2a were produced by AS of the NAC transcription factor PtATAF 1.2 in *P. trichocarpa*. The tolerance of transgenic plants overexpressing PtATAF1.2b to drought stress was significantly higher than that of transgenic plants overexpressing PtATAF1.2a [21]. The nac5 domain and nac125 domain, truncated variable splicing bodies in maize, act as dominant negatives that interfere with the corresponding full-length ZMNAC5 and ZMNAC125 proteins, respectively, weakening the role of NACs in the tolerance of Cd [22]. In *Populus tomentosa*, a naturally occurring alternative splicing variant (intron retention, IR) of PtRD26, PtRD26IR, was identified and shown to be a repressor of leaf senescence via its dominant negative effect on PtRD26 and another hub Sen-NAC TFs [23].

For moso bamboo, 152 PeNAC genes have been predicted [24]. Some NAC genes (*PeNAC1*, *PheNAC1*, *PheNAC3*, *PeSNAC-1*) are involved in the drought and salt stress response of moso bamboo [24,25,26,27], while the functions of other NAC genes have not been thoroughly characterized in moso bamboo. Transcriptome sequencing of the development process of moso bamboo flowers revealed that the *PH01000110G0680* (*PheNAC23*) gene, which belongs to the NAC transcription factor family, is up-regulated during flower development [28], suggesting its potential involvement in the flowering and senescence process of moso bamboo. Further research indicates that alternative splicing events occur in the *PheNAC23* gene during leaf development, in leaves during flower development, and under drought stress treatment.

Considering the crucial role of NACs in mediating stress and flowering, as well as the significant function of AS in TF functionality, in this study, we isolated a stress-responsive NAC transcription factor (PheNAC23) and its splicing variant, PheNAC23^ES^. The amino acid (aa) sequences of PheNAC23 had 87% and 67% similarity to PheNAC1 (named *PheNAC105* in our study) and PheNAC3 protein (named *PheNAC38* in our study), respectively. The relative expression of *PheNAC23^ES^* was different from *PheNAC23* during the natural senescence of leaves and drought stress treatment in the leaves of moso bamboo. Overexpression of *PheNAC23* and *PheNAC23^ES^* exhibited opposing effects on flowering regulation and drought tolerance in transgenic Arabidopsis plants, suggesting the function of PheNAC23^ES^ might be opposite to PheNAC23. Finally, PheNAC23^ES^ had no transactivation activity but could form a heterogenous complex with PheNAC23. Therefore, PheNAC23^ES^ might serve as a small interfering peptide that affects the function of PheNAC23.

## 2. Results

### 2.1. Identification and Phylogenetic Analysis of PheNACs

A total of 235 nonredundant *PheNAC* genes (*PheNACs*) were identified in the moso bamboo genome. Among them, 141 *PheNACs* contained a complete NAC domain, and the other 94 *PheNACs* had an incomplete NAM domain. PheNAC proteins with lengths of 74 to 907 aa residues possess a predicted molecular mass ranging from 8.22 to 99.31 kDa and a theoretical isoelectric point (pI) of 4.41–10.56 (Supplementary Table S1). Among the PheNAC proteins, fifteen PheNACs were predicted to contain α-helical transmembrane, recognized as membrane-bound transcription factors.

**Figure 1:**
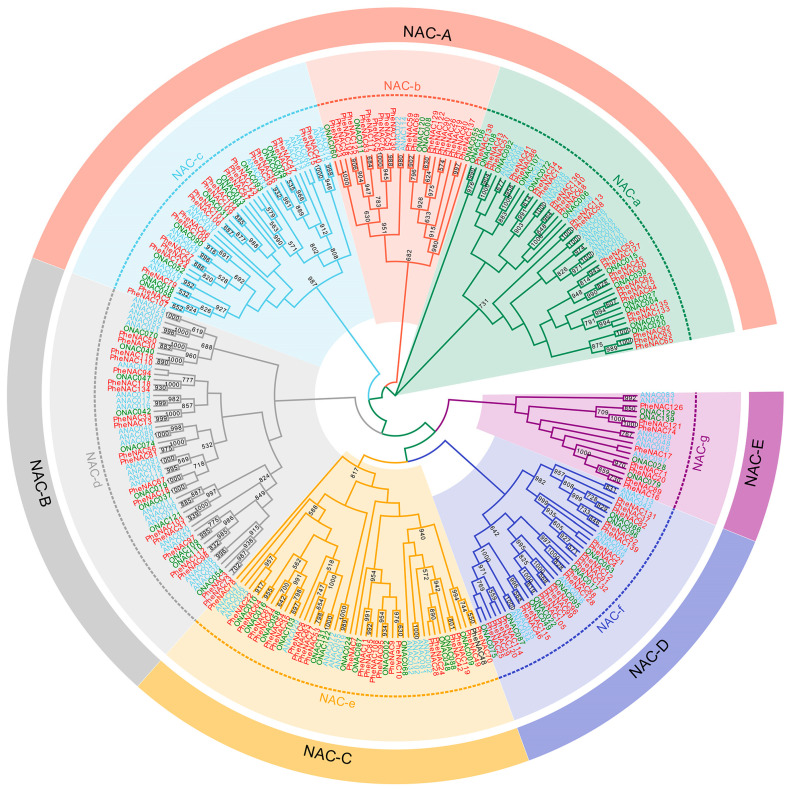
Phylogenetic analysis of the NAC transcription factors of moso bamboo, Arabidopsis and rice. NJ phylogeny of 278 NAC genes of three species, as determined by MEGA 7.0. The colored shading marks the subgroups of the NACs. Numbers on branches are bootstrap proportions from 1000 replicates.

To determine the evolutionary relationship of NAC genes among moso bamboo, Arabidopsis and rice, the conserved domains of 279 NAC genes (141 PheNACs of moso bamboo, 75 *AtNACs* of Arabidopsis, 64 *OsNACs* of rice) with complete NAM domains were subjected to multiple sequence alignment. According to the results of Fang et al. [29], The NACs were classified into five groups (NAC-A–NAC-E) or seven subgroups (NAC-a–NAC-g) based on the topology of the neighbor-joining (NJ) tree constructed by MEGA 7 (Figure 1). The NAC-A group, with the most significant number of 118 members, comprised the NAC-a, NAC-b, and NAC-c subgroups. Beyond that, each group comprised only one subgroup, namely, NAC-B/NAC-d, NAC-C/NAC-e, NAC-D/NAC-f, and NAC-E/NAC-g. The low support rate of these subgroups was expected for the NAC family due to the variable transcriptional regulation region at the C-terminus, as shown in previous research [29]. However, low-level branches showed relatively high bootstrap values, indicating the conservative evolution of subgroup genes.

To examine the evolutionary relationship of PheNACs, NJ trees of PheNACs (including proteins with an incomplete NAM domain) were also constructed using the full-length PheNAC proteins (Supplementary Figure S1). PheNACs were classified into seven subgroups based on the evolution of PheNACs with a complete NAM domain. The NAC-b, NAC-c, and NAC-e subgroups were clustered with independent branches with high support over 50%. The majority of members of these three subgroups had a complete NAM domain. In contrast, more PheNACs with incomplete NAM domains were mainly found in the subgroups of NAC-a, NAC-d, NAC-f, and NAC-g, especially for NAC-d and NAC-g, which resulted in irregular evolutionary relationships among NAC-d and NAC-g and NAC-f.

### 2.2. PheNAC23 Locus Expressed Two Distinct Transcripts

**Figure 2:**
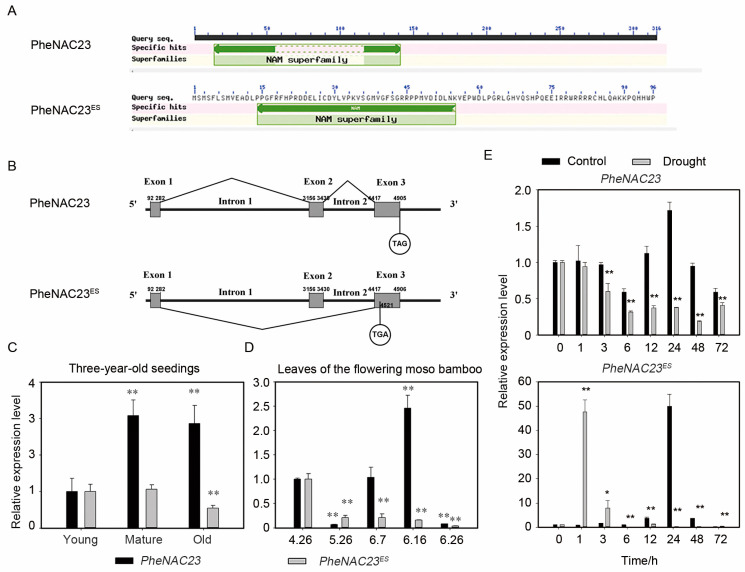
The analysis of NAM domain, intron/exon structure and expression patterns of *PheNAC23* and *PheNAC23^ES^*. (**A**) NAM domain analysis; (**B**) The intron/exon structure of *PheNAC23* and *PheNAC23^ES^*; (**C**) Expression patterns of *PheNAC23* and *PheNAC23^ES^* in leaves of three-year-old seedlings; (**D**) Expression patterns of *PheNAC23* and *PheNAC23^ES^* in leaves of the flowering moso bamboo; 4.26–6.26: dates of sample collection (26 April, 26 May, 7 June, 16 June, and 26 June); 4.26: floral bud formation stage; 5.26 and 6.7: inflorescence growing stage; 6.16: bloom stage; 6.26: embryo formation stage. (**E**) Expression levels of *PheNAC23* and *PheNAC23^ES^* under PEG treatment. The relative expression at 0 h was normalized to 1; values represent the mean ± SE from three biological replicates. * *p* < 0.05, ** *p* < 0.01.

Based on the evolutionary relationship of *NACs*, we observed four *PheNACs* grouped with *ONAC060* and *ONAC011* of rice, which have been demonstrated as stress and/or senescence-related factors (Figure 1). Previously, we verified that *PheNAC38* and *PheNAC125* were positive and negative drought/salt tolerance regulators [25,27], and *PheNAC23* is up-regulated during flower development according to the transcriptome sequencing of the development process of moso bamboo flowers [28]. In this study, the coding sequence (CDS) sequence of the *PheNAC23* gene was isolated. When the leaves of three-year-old seedlings, six-month-old seedlings, flowering moso bamboo at different stages, and leaves under drought stress were used as templates, two distinct transcripts were generated from RT-PCR (reverse transcription polymerase chain reaction) (Supplementary Figure S2). The larger sequence (951 bp) corresponded to the annotated PheNAC23 protein in the moso bamboo genome, while the smaller sequence (656 bp) encoded a protein with an incomplete NAM domain (see Figure 2A). After aligning the transcripts to the genome, we confirmed that both PheNAC23 transcripts originated from the same genetic locus. The shorter variant, referred to as *PheNAC23^ES^*, resulted from the exclusion of Exon 2 during splicing (Figure 2B).

To evaluate the expression patterns of *PheNAC23* and *PheNAC23^ES^* under stress- and senescence-related conditions, the expressions of *PheNAC23* and *PheNAC23^ES^* were detected in different developmental leaves of three-year-old seedings (Figure 2C). According to Ren et al. [30], we divided bamboo leaves into young, mature, and senescent stages. We observed that *PheNAC23* accumulated after leaf maturity and persisted through senescence, while *PheNAC23^ES^* was downregulated during leaf senescence (Figure 2C). Furthermore, we detected their expression in the leaves of flowering moso bamboo (Figure 2D). Moso bamboo flowering typically coincides with a change in the physiological status of bamboo and is often seen as a sign of stress-induced aging or natural decline in bamboo forests [3]. *PheNAC23* was upregulated after flowering, especially during the blooming stage (Figure 2D). In contrast, *PheNAC23^ES^* was constantly suppressed in the leaves of flowering bamboo. For the PEG-modeled drought stress treatments, the relative expression level of the *PheNAC23* gene was lower than that of the control group (Figure 2E), while *PheNAC23^ES^* had significantly upregulated expression after 1 h and 3 h of drought treatment. In summary, *PheNAC23* was down-regulated under drought-related conditions and upregulated after bamboo flowering, conditions which might lead to individual senescence. Conversely, *PheNAC23^ES^* showed the opposite expression pattern in senescent leaves and leaves during the flowering duration. Therefore, we supposed that *PheNAC23* and *PheNAC23^ES^* might have opposite roles in the senescence and drought responses of moso bamboo.

### 2.3. PheNAC23 and PheNAC23^ES^ Had Different Subcellular Localization and Transcriptional Activity

**Figure 3:**
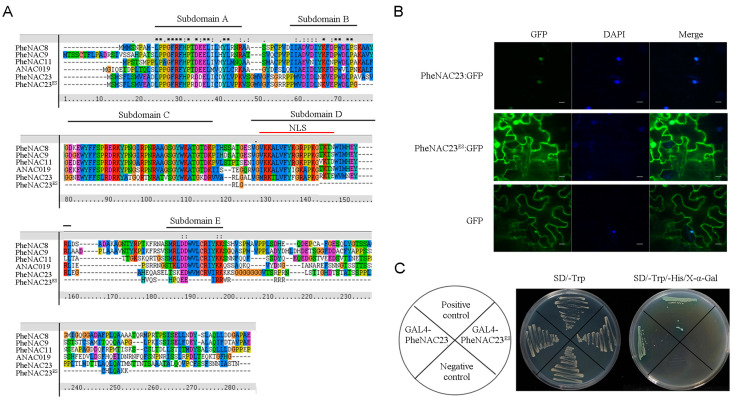
Characterization of PheNAC23 and PheNAC23^ES^ protein sequences, subcellular localization and transcriptional activation analysis. (**A**) The multiple sequence alignment of four PheNACs belonging to the NAP subgroup, ANAC019 in Arabidopsis, PheNAC23 and PheNAC23^ES^; NLS: nuclear localization signal. (**B**) Confocal images of subcellular localizations of PheNAC23 and PheNAC23^ES^ in *Nicotiana benthamiana* (tobacco) leaf epidermal cells; expression of the PheNAC23-GFP and PheNAC23^ES^-GFP fusion protein was examined after 3 d by fluorescence and light microscopy; 4′,6-diamidino-2-phenylindole (DAPI) was used to stain the cell nucleus; bar, 20 μm. (**C**) Transcriptional activation analysis of PheNAC23 and PheNAC23^ES^ fused with the GAL4 DNA-binding domain in yeast. The positive control is pGBKT7-53 + pGADT7-T; the negative control is the empty pGBKT7 vector. X-α-Gal tests the expression of α-galactosidase.

To test our hypothesis, we first analyzed the domains of PheNAC23 and PheNAC23^ES^ based on the protein structure characteristics of ANAC019 [31]. The results showed that PheNAC23 contained complete A–E subdomains, but PheNAC23^ES^ had only solitary subdomain A, which was proven as the essential domain to form the dimer (Figure 3A). Highly conserved C and D subdomains involved nuclear localization and DNA binding [5,6]. Therefore, the nuclear localization and DNA-binding capabilities might be deficient in PheNAC23^ES^. We further examined the subcellular localization of the PheNAC23 and PheNAC23^ES^ proteins. The results indicated that PheNAC23 was found in the nucleus, while PheNAC23^ES^ was located in the nucleus and cytoplasm (Figure 3B). The prematurely terminated PheNAC23^ES^ lost the nuclear localization signal peptides. Additionally, the transcriptional activity of PheNAC23 and PheNAC23^ES^ was performed in a yeast reporter system. PheNAC23 activated the expression of the *His-3* and *β-Gal* solidly, which indicated that it should be a transcription activator. However, PheNAC23^ES^ failed to activate any reporters of *His-3* and *β-Gal* (Figure 3C). These results suggested that PheNAC23^ES^ might have other functions than the nucleus-located PheNAC23, which functions as a typical TF in the nucleus.

### 2.4. PheNAC23^ES^ Formed a Heterodimer with PheNAC23

**Figure 4:**
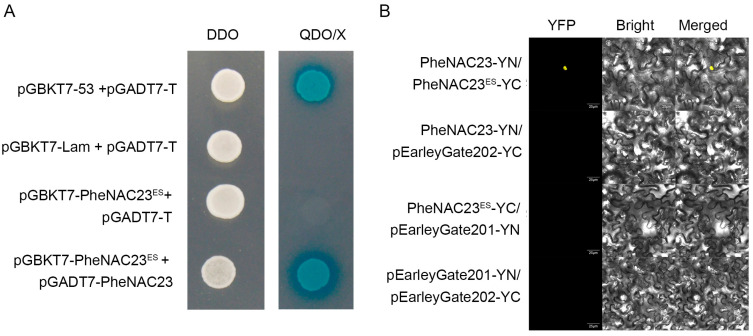
PheNAC23^ES^ interacts with PheNAC23. (**A**) Yeast cells of co-transformants of PheNAC23 and PheNAC23^ES^ grown on synthetic dropout medium without tryptophan and leucine (SD/-Trp-Leu) and SD/-Trp-Leu-Ade (Adenine)-His (Histidine) + X-α-gal medium at 30 °C for 3 d. PheNAC23^ES^ was fused to the DNA-binding domain (BD), and PheNAC23 was fused to the transcription activation domain (AD). pGBKT7-53 and pGADT7-T are positive controls. pGBKT7-Lam and pGADT7-T are negative controls. (**B**) BiFC assay reveals the interaction between PheNAC23^ES^ and PheNAC23. PheNAC23 was fused to pEarleyGate201-YN, and PheNAC23^ES^ was fused to pEarleyGate202-YC. *Agrobacteria* carrying different plasmids, as indicated, were co-expressed in tobacco. Representative images of tobacco leaves 48 h after infiltration are shown. Scale bar: 25 μm.

It was reported that NAC proteins can function as homodimers or heterodimers in plant cells [7]. Therefore, we explored whether PheNAC23^ES^ can function as the heterodimer of PheNAC23. We found that PheNAC23^ES^ could interact with PheNAC23 by yeast two-hybrid (Y2H) assay (Figure 4A). The protein–protein interaction of PheNAC23^ES^ and PheNAC23 was further verified by the bimolecular fluorescence complementation (BiFC) assay in the tobacco transient transformation system. The results of the BiFC assay showed that YFP signals could be detected in the nucleus after transformation of PheNAC23 and PheNAC23^ES^, whereas YFP signals could not be detected in the nucleus of control experimental group cells (Figure 4B), so PheNAC23^ES^ formed a heterodimer with PheNAC23.

### 2.5. PheNAC23 and PheNAC23^ES^ Have Opposite Functions in Regulating Flowering

**Figure 5:**
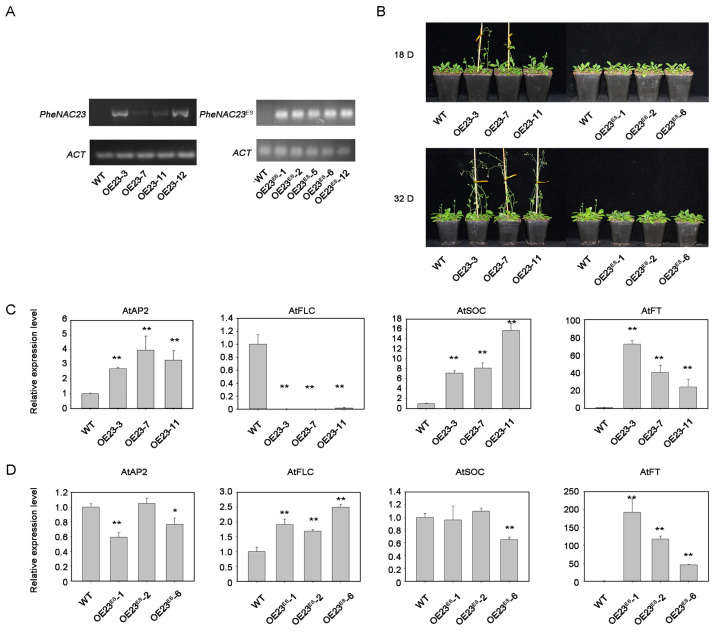
Overexpression of *PheNAC23* and *PheNAC23^ES^* promotes/delays flowering in Arabidopsis. (**A**) RT-PCR confirmed the relative expression levels of *PheNAC23* and *PheNAC23^ES^* in overexpression lines. OE23-3, OE23-7, OE23-11 and OE23-12 were four independent lines that overexpressed *PheNAC23*. OE23^ES^-1, OE23^ES^-2, OE23^ES^-5, OE23^ES^-6 and OE23^ES^-12 were five independent lines that overexpressed PheNAC23^ES^. (**B**) Morphology of Arabidopsis plants overexpressing *PheNAC23* and *PheNAC23^ES^* at flowering stage. (**C**,**D**) Analysis of relative expression levels of flowering-related genes in transgenic Arabidopsis lines and WT. Asterisks indicate that the value is significantly different from that of the WT at the same time point (* *p* < 0.05, ** *p* < 0.01).

To further investigate the functions of *PheNAC23* and *PheNAC23^ES^*, *PheNAC23* and *PheNAC23^ES^* were overexpressed in Arabidopsis. An RT-PCR assay confirmed that *PheNAC23* and *PheNAC23^ES^* exhibited high expression levels in the overexpression lines (Figure 5A). The *PheNAC23*-overexpression lines flowered substantially earlier than wild type (WT), while the *PheNAC23^ES^*-overexpression lines flowered later than *PheNAC23* and WT (Figure 5B). This demonstrated the opposite functions of *PheNAC23* and *PheNAC23^ES^* in regulating flowering. Furthermore, the floral repressors and activators (*AtAP2*, *AtFT*, *AtFLC*, and *AtSOC*) of Arabidopsis were detected in transgenic lines (Figure 5C). In the *PheNAC23*-overexpression line, the expression levels of flowering-promoting genes *AtAP2*, *AtFT*, and *AtSOC* were up-regulated, and that of the flowering-related negative regulatory gene *AtFLC* was down-regulated. However, in the *PheNAC23^ES^*-overexpression lines, the flowering-related negative regulatory gene *AtFLC* was up-regulated.

### 2.6. PheNAC23 and PheNAC23^ES^ Have Opposite Functions in Drought Stress Response

**Figure 6:**
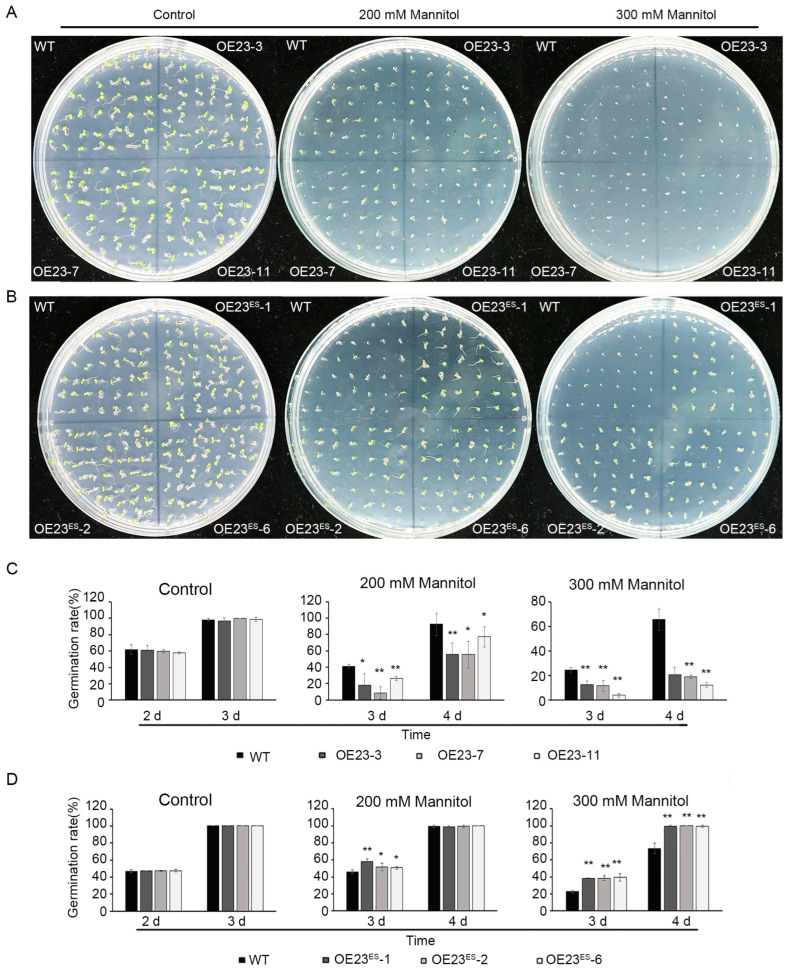
Seed germination of *PheNAC23-* and *PheNAC23^ES^*-overexpressing Arabidopsis under mannitol. (**A**,**B**) Photographs were taken 4 days after mannitol treatment. (**C**,**D**) The germination rate was determined at 2–4 days after mannitol treatment. WT, wild-type Arabidopsis; OE23-3, OE23^ES^-7, and OE23^ES^-11, three independent lines overexpression of *PheNAC23^ES^*; OE23^ES^-1, OE23^ES^-2, and OE23^ES^-6, three independent lines overexpression of *PheNAC23^ES^*; The experiments were repeated three times with similar results. Asterisks indicate the significant difference between WT and *PheNAC23-* or *PheNAC23^ES^* -overexpressing Arabidopsis, * *p* < 0.05 and ** *p* < 0.01); error bars indicate the ± SE.

**Figure 7:**
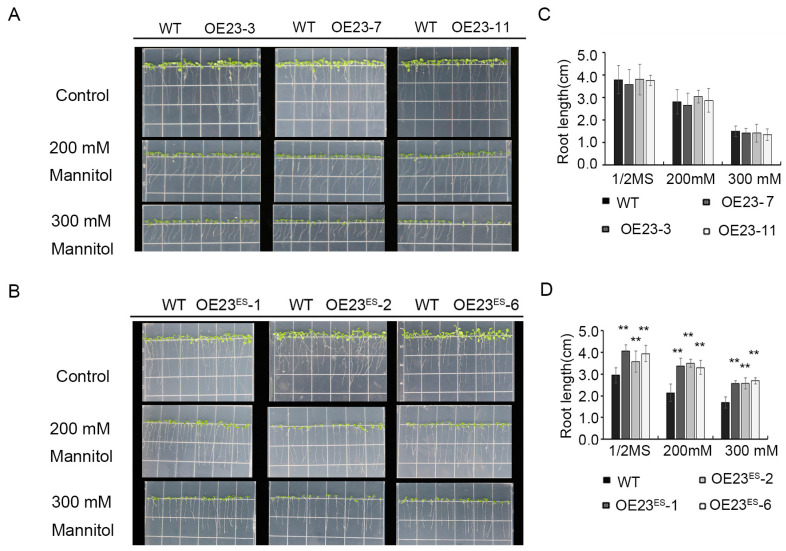
Phenotypic analysis of roots of PheNAC23- and PheNAC23^ES^-overexpressing Arabidopsis under mannitol treatment. (**A**,**B**) Photographs of taproot length were taken 10 days after mannitol treatment, each square measures 1.5 × 1.5 cm. (**C**,**D**) Statistical analysis of taproot length under mannitol treatment. WT, wild-type Arabidopsis; OE23-3, OE23-7, and OE23-11 are three independent lines of *PheNAC23*; OE23^ES^-1, OE23^ES^-2, and OE23^ES^-6 are three independent lines overexpressing *PheNAC23^ES^*. Asterisks indicate a significant difference between WT and *PheNAC23-* or *PheNAC23^ES^*-overexpressing Arabidopsis; ** *p* < 0.01; error bars indicate the ±SE.

The germination and growth of the WT, *PheNAC23*-overexpression and *PheNAC23^ES^*-overexpression lines were surveyed on 1/2 Murashige and Skoog medium (MS) with additional amounts of mannitol (0 mM, 200 mM and 300 mM), simulating drought stress (Figure 6 and Figure 7). On the third day after germination, no significant difference in the germination rate between the WT and transgenic lines was observed in the control groups (1/2 MS with 0 mM mannitol). However, after exposure to 200 mM mannitol stress, the germination rate of *PheNAC23*-overexpression lines was markedly decreased compared to WT on the third and fourth days after germination (Figure 6A,C). In contrast, the *PheNAC23^ES^*-overexpression lines exhibited higher germination rates than WT on the third day and completely germinated on the fourth day, similar to WT. After treatment with a higher concentration of 300 mM mannitol, the *PheNAC23^ES^*-overexpression lines consistently exhibited higher germination rates than WT on the third and fourth day (Figure 6B,D), while the PheNAC23-overexpression lines displayed a much lower rate.

A root length phenotype was surveyed ten days after planting the seedlings on the medium (Figure 7). The average taproot lengths of *PheNAC23^ES^*-overexpression lines were 3.95 ± 0.04 cm, 3.39 ± 0.04 cm, and 2.61 ± 0.04 cm on 1/2 MS supplied with 0 mM, 200 mM and 300 mM mannitol, respectively, which was significantly longer than for WT (Figure 7B,D). Although the root length of the *PheNAC23*-overexpression lines was inhibited by mannitol treatment, it was comparable to WT (Figure 7A,C). This indicated that *PheNAC23^ES^* improved the drought tolerance of Arabidopsis, while *PheNAC23* did not, even adversely affecting the seed germination under stress conditions.

## 3. Discussion

The NAC family is one of the most prominent TF families in plants and plays essential roles during developmental processes and stress responses. The specific functions of most NAC genes still need further investigation, especially for moso bamboo, an economically vital resource in Asia. In this study, we identified 235 *PheNAC* genes with the NAM domain (PF02365), in which 151 genes were identical to the previous report [24]. The other 84 genes were newly identified in this study (Table S1). Although 94 *PheNACs* recorded proteins with a partial NAM domain, we documented them in this study because these proteins with imperfect NAM may have a potential function related to NAC proteins, which was proved previously [19,21,22,23]. Our preliminary study of PheNAC23 and PheNAC23*^ES^* also supported this point of view.

Highly conserved genes tend to have more AS events than poorly conserved genes, consistent with the trend that conserved genes are apt to have higher connectivity in gene–gene interaction networks [32,33,34,35,36]. AS events improve the flexibility of molecular regulation at the post-transcriptional level and enrich the protein diversity from highly conserved inherited loci. The proteins produced from the same genetic locus always have similar biological functions during plant development. In *Populus tomentosa*, *PtRD26IR* was generated from an intron-retention (IR) event, which retained the protein dimerization domain but lacked the DNA-binding domain. PtRD26IR was shown to be a repressor of leaf senescence via its dominant-negative effect on PtRD26 and other hub Sen-NAC TFs [23]. This study generated two isoforms from the same locus of PH02Gene07625, PheNAC23 and PheNAC23^ES^. PheNAC23^ES^ only contains highly conserved subdomain A and non-conserved subdomain B, thus retaining the protein dimerization domain but lacking the DNA-binding domain. During leaf senescence, flower development and drought stress, *PheNAC23^ES^* showed a different expression pattern from *PheNAC23*. The subcellular localization and transcriptional activation activity prediction results showed that PheNAC23 was localized in the nucleus and had transcriptional activation activity, but PheNAC23^ES^ had similar localization to the empty vector due to the absence of nuclear localization signals and did not have transcriptional activation activity. This indicates the different roles of PheNAC23^ES^ and PheNAC23 in regulating the senescence and drought stress response of moso bamboo.

The NAC transcription factor gene *OsY37* (*ONAC011*) in rice promoted leaf senescence and accelerated heading time [14]. Overexpression of *PheNAC3* promoted leaf senescence and enhanced the drought resistance of *A. thaliana* [25]. *PheNAC23* belongs to the same subfamily as *ONAC011* and *PheNAC3* (named *PheNAC125* in this study) (Figure 1). Similar to the positive role of ONAC011 in leaf senescence and heading time in rice, *PheNAC23* accelerated flowering in Arabidopsis. The lines with overexpression of *PheNAC23^ES^* showed a later-flowering phenotype. Moreover, *PheNAC23^ES^* enhanced the drought tolerance of Arabidopsis, while PheNAC23 reduced the drought tolerance. These results indicate that *PheNAC23* may promote flowering and play a negative regulatory role in drought stress, while *PheNAC23^ES^* has the opposite function.

In Arabidopsis, the *AtFT* gene moves from the leaf to shoot apex and induces flowering. GALACTINOL SYNTHASE promoter (GAS1):FT complemented the ft-7 mutation, and the transgenic plants flowered earlier than did wild-type plants [37]. FLOWERING LOCUS C (FLC) is a major repressor of flowering in Arabidopsis that binds and represses the *AtFT* and *AtSOC1* genes [38,39]. AGL20 (SOC1) acts as a floral activator and is repressed by the interaction of FRI and FLC in Arabidopsis [40]. In this context, the *AtFT* and *AtSOC1* genes were significantly induced, and *AtFLC* was repressed in *PheNAC23* transgenic lines. *AtSOC1* gene was repressed, and *AtFLC* was induced in *PheNAC23 ^ES^* transgenic lines. These differences suggest that the functional mechanisms of *PheNAC23* and *PheNAC23^ES^* in flowering were different. We proposed that this gene might participate in flowering regulation by regulating the expression of the flowering-related genes *AtSOC1* and *AtFLC.*

This study found that the truncated PheNAC23^ES^ formed heterodimers with PheNAC23. Similar formations have been reported in other plants. The isoform OsCYL4b of rice displayed distinct protein location and stress functions compared with the alternative spliceosome OsCYL4a. It was suggested to be a negative inhibitor of the cyclization of OsCYL4a, promoting abscisic acid (ABA) production to regulate stomatal closure or H_2_O_2_ generation [41]. In poplar, PtRD26IR formed heterodimers with PtRD26 and other NACs, inhibiting their DNA-binding activity and delaying leaf senescence [23]. Considering the interactions between PheNAC23^ES^ and PheNAC23 and their opposite functions, we speculated that PheNAC23^ES^ might influence PheNAC23 function by forming a heterodimer. And, of course, the truncated PheNAC23^ES^ may also form heterodimers with other NAC proteins, which needs further investigation. These findings provided fundamental information for a better understanding of the function of *PheNAC23* in moso bamboo and offer candidate NAC genes for further studies on their roles in flowering, senescence and stress responses.

## 4. Materials and Methods

### 4.1. Identification of PheNACs in Moso Bamboo and Other Species

The annotated NAC genes of Arabidopsis and Rice were obtained from the Arabidopsis Information Resource (http://www.arabidopsis.org/, accessed on 6 April 2020) and the Rice Genome Annotation Project (http://rice.uga.edu/, accessed on 10 January 2020). The NAC protein sequences of these plants were used as query sequences to search the moso bamboo Genome Database (BambooGDB, http://gigadb.org/dataset/100498, accessed on 10 January 2020) with an e-value of 10^−10^. The obtained putative sequences were subjected to reblast of the protein database with an e-value of 10^−5^. The putative PheNACs were annotated to the NCBI NR database with an e-value cutoff of 10^−10^ using Blast2GO [42]. Then, the NAM domain (PF02365) of the putative PheNACs was manually checked by CD-search (https://www.ncbi.nlm.nih.gov/Structure/cdd/wrpsb.cgi, accessed on 25 October 2021).

### 4.2. Phylogenetic Tree Construction and Sequence Analysis

Multiple alignments of PheNACs were performed using the ClustalX program with default settings [43]. The NJ tree was constructed using MEGA 7 with the bootstrap assessment of 1000 replicates.

The NAC full-length mRNA and genomic DNA sequences were retrieved from the moso bamboo database. The intron/exon structure of the NAC genes was determined using Gene Structure Display Server (http://gsds.cbi.pku.edu.cn/, accessed on 25 October 2021) [44]. The NAC protein pI and Mw were determined by the Compute pI/Mw tool (http://web.expasy.org/compute_pi/, accessed on 25 October 2021). The conserved motifs were identified using MEME version 5.0.3 (https://meme.nbcr.net/, accessed on 25 October 2021) with the following parameters: number of repetitions (any), maximum number of motifs (20), and the optimum motif widths were constrained to between 6 and 200 residues [45]. The predictions of membrane-bound proteins were determined by TMHMM server v.2.0 (https://dtu.biolib.com/DeepTMHMM, accessed on 25 October 2021).

### 4.3. Plant Material and Treatments

Moso bamboo seeds were collected from Dajing County, Guilin (E 110°17′–110°47′; N 25°04′–25°48′) in the Guangxi Zhuang Autonomous Region. Leaves of flowering moso bamboo at different stages were collected according to the methods described previously for quantitative reverse transcription polymerase chain reaction (qRT–PCR) experiments [28]. The sampling dates were 26 April, 26 May, 7 June, 16 June, and 26 June, corresponding to the following stages: 26 April—floral bud formation stage; 26 May and 7 June—inflorescence growth stage; 16 June—bloom stage; and 26 June—embryo formation stage.

Moso bamboo seedlings were grown in a greenhouse at 22 °C under long-day conditions (16 h light/8 h dark) and watered in 1/2 strength Hoagland’s nutrient medium once a week.

The different developmental stages of leaves of moso bamboo seedlings were sampled according to Ren et al. [30]. The moso bamboo culm branches have clusters of 5–7 leaves at the branch tips. The leaves are arranged alternately, with older leaves (L1) closer to the branch point and younger leaves (L7) farther away. As the transition occurs between old and young leaves, the older leaves (L1–L4) gradually senesce, showing signs such as chlorosis, yellowing, and wrinkling, eventually drying out and falling off. The mature leaves (L5–L6), located between the old and young leaves, are dark green, flat, and relatively large in size. In contrast, the young leaves (L7) are lighter in color and softer in texture. Based on these characteristics, young leaves, mature leaves, and senescent leaves of three-year-old and six-month-old seedlings were collected for gene cloning and qRT–PCR experiments.

Two-month-old moso bamboo seedlings were subjected to abiotic stress treatments. The seedlings were watered with polyethylene glycol 6000 (PEG 6000) solution (20%, *m*/*v*) to mimic drought stress. The control seedlings were obtained without any stress treatment. The seedlings’ second and third young leaves were sampled at 0 h, 1 h, 3 h, 6 h, 12 h, 24 h, 48 h, and 72 h after abiotic stress treatments for gene cloning and qRT–PCR experiments.

### 4.4. Gene Expression Analysis PheNAC23 and PheNAC23^ES^

For qRT–PCR, Primer 3 web version 4.1.0 (http://primer3.ut.ee/, accessed on 25 April 2022) was used to design the specific primers according to the *PheNAC* gene sequences (Supplementary Table S2). Data acquisition and analyses were performed by Roche Light Cycler software on a Light Cycler 480 (Roche, Rotreuz, Switzerland). According to the manufacturer’s instructions, the 20 μL reaction mixture contained 0.4 μL (10 μM) of each primer, 2 μL of cDNA, 10 μL of SYBR Green I Master Mix, and 7.2 μL ddH_2_O. *TIP41* (tonoplast intrinsic protein 41) was selected as an internal control [46]. qRT–PCR assays were performed with three biological and three technical replicates. Quantitative analysis was performed using 2^−ΔΔCT^ [47].

### 4.5. PheNAC23 Gene Cloning, Subcellular Localization and Transcriptional Activation

The full-length coding region of *PheNAC23* was amplified in the leaf tissue of moso bamboo using gene-specific primers (Supplementary Table S2) and Takara LA Taq DNA Polymerase (Takara Biomedical Technology, Shiga, Japan). PCR products were cloned into pGEM^®^-T Easy vector (Promega, Madison, WI, USA) and verified by GENEWIZ (Suzhou, China) through sequencing.

The subcellular localization of PheNAC23 and PheNAC23^ES^ was performed through a tobacco leaf transient expression system [48]. Full-length *PheNAC23* and *PheNAC23^ES^* were ligated into the pCAMBIA2300-35S-eGFP vector and fused in frame with GFP. After a 3-day post-infiltration period, a confocal laser scanning microscope examined the fluorescence signals in transfected leaves (Zeiss LSM 5 Live).

For transcriptional activation analysis, full-length *PheNAC23* and *PheNAC23^ES^* were fused in a frame with the GAL4 DNA-binding domain in pGBKT7 (Takara Biomedical Technology, Shiga, Japan). The recombinant vectors and the pGBKT7 empty vector (control) were transferred into yeast strain AH109 using lithium acetate. The transformed strains were cultured on a minimal medium (Coolaber, Beijing, China) without His and Trp and assayed for α-galactosidase activity and growth status.

### 4.6. Overexpression of PheNAC23 and PheNAC23^ES^ in Arabidopsis

The coding sequences of *PheNAC23* and *PheNAC23^ES^* were cloned into the pCAMBIA 2300 vector according to Cui et al. [49]. The recombinant vectors pCAMBIA2300-PheNAC23 and pCAMBIA2300-PheNAC23^ES^ were introduced into *Agrobacterium tumefaciens* strain GV3101 for Arabidopsis transformation into Arabidopsis plants (Columbia-0) through the floral dipping method [50]. Transgenic plants were selected on ½ MS solid media supplemented with 50 mg L^−1^ kanamycin. The T_3_ generation was selected to examine flowering time under a 16 h light/8 h dark cycle at 22 °C. The leaves were harvested and used for qRT–PCR experiments.

Transgenic Arabidopsis seeds were sown on ½ MS medium solid plates with 200 mM or 300 mM mannitol to mimic drought stress, stratified at 4 °C for 2 days, and then transferred to long-day growth conditions (16 h light/8 h dark cycle at 23 ± 2 °C) in a growth chamber. The germination rate of transgenic Arabidopsis seeds was analyzed and recorded after 2, 3, and 4 days. The taproot lengths were measured after 10 days.

### 4.7. Y2H and BiFC Assays

Y2H assays using the Matchmaker GAL4 two-hybrid system (Clontech, Beijing, China) were performed according to the manufacturer’s instructions. The coding sequence of PheNAC23^ES^ was cloned into pGBKT7, and the coding sequence of PheNAC23 was cloned into pGADT7, while pGBKT-53 + pGADT7-T and pGBKT-53 + pGADT7-Lam were the positive and negative controls, respectively. The plasmids were co-transformed into AH109 yeast stains, and positive transformants containing pGBKT7-PheNAC23^ES^ and pGADT7-PheNAC23 plasmids were selected through SD/-Leu/-Trp medium. Subsequently, the interaction between PheNAC23^ES^ and PheNAC23 was analyzed based on the growth of the positive transformants on SD/-Leu/-Trp/-His/-Ade medium.

For BiFC assays, the coding sequences of *PheNAC23* and *PheNAC23^ES^* were fused in pEarleyGate201-YN and pEarleyGate202-YC, respectively. All empty vectors were used as negative controls, and YN-PheNAC23 and PheNAC23^ES^-YC were also co-transformed with the pEarleyGate201-YN or pEarleyGate202-YC vector to exclude a false positive. These constructs were transient expressions in tobacco leaf, as described in [48]. The transfected leaves were imaged by confocal microscopy with a 510 nm argon laser. The combinations of BiFC assays involved at least three biological replicates.

## Data Availability

The original contributions presented in the study are included in the article/Supplementary Materials, further inquiries can be directed to the corresponding author.

## Supplementary Materials

The following supporting information can be downloaded at https://www.mdpi.com/article/10.3390/plants13233452/s1. Figure S1: Phylogenetic relationships of the moso bamboo NAC genes; Figure S2: Cloning of the CDS of the PheNAC23; Table S1: Nomenclature and Classification of NAC family genes in moso bamboo; Table S2: The list of primers.
