# Supplementary material for: Alternative Splicing of PheNAC23 from Moso Bamboo Impacts Flowering Regulation and Drought Tolerance in Transgenic Arabidopsis
Source: Plants (Basel). 2024 Dec 9;13(23):3452. doi: 10.3390/plants13233452 (PMC11644316; doi:10.3390/plants13233452)
Supplement: Supplementary file 1 [file plants-13-03452-s001.zip › plants-3289207-Supplementary Materials.pdf]

Table S1. Nomenclature and Classification of NAC family genes in moso bamboo.

Gene name	Gene ID	Scaffords	Number (aa)	MW (kDa)	Isoelectric points	subcellular localization	MTFs
PheNAC1	PH02Gene00821.t1	scaffold_21	302	33.39	7.76	Nucleus.	
PheNAC2	PH02Gene00868.t1	scaffold_21	351	39.10	5.19	Nucleus.	
PheNAC3	PH02Gene00916.t1	scaffold_21	658	73.42	5.63	Nucleus.	
PheNAC4	PH02Gene01045.t1	scaffold_17	393	44.17	8.89	Nucleus.	
PheNAC5	PH02Gene01804.t1	scaffold_23	287	31.51	6.1	Nucleus.	
PheNAC6	PH02Gene02053.t1	scaffold_24	317	36.60	6.15	Nucleus.	
PheNAC7	PH02Gene02411.t1	scaffold_7	247	27.97	7.05	Nucleus.	
PheNAC8	PH02Gene03448.t1	scaffold_12	361	40.39	6.32	Nucleus.	
PheNAC9	PH02Gene03725.t1	scaffold_14	296	33.51	6.01	Nucleus.	
PheNAC10	PH02Gene04473.t1	scaffold_16	288	32.62	8.69	Nucleus.	
PheNAC11	PH02Gene04543.t1	scaffold_10	268	30.25	5.77	Nucleus.	
PheNAC12	PH02Gene04571.t1	scaffold_22	380	42.55	6.54	Nucleus.	
PheNAC13	PH02Gene04645.t1	scaffold_18	629	69.04	4.71	Nucleus.	yes
PheNAC14	PH02Gene04659.t1	scaffold_18	348	38.65	9.33	Nucleus.	
PheNAC15	PH02Gene05167.t1	scaffold_23	336	36.92	6.65	Nucleus.	
PheNAC16	PH02Gene05211.t1	scaffold_23	201	22.36	5.03	Nucleus.	
PheNAC17	PH02Gene05304.t1	scaffold_6	416	46.99	5.2	Nucleus.	
PheNAC18	PH02Gene05356.t1	scaffold_22	690	76.85	4.72	Nucleus.	yes
PheNAC19	PH02Gene05850.t1	scaffold_17	293	32.87	6.56	Nucleus.	
PheNAC20	PH02Gene06239.t1	scaffold_3	205	22.60	5.96	Nucleus.	
PheNAC21	PH02Gene06771.t1	scaffold_18	405	45.37	8.19	Nucleus.	
PheNAC22	PH02Gene06980.t1	scaffold_10	415	44.27	7.78	Nucleus.	
PheNAC23	PH02Gene07625.t1	scaffold_11	316	34.48	9.08	Nucleus.	
PheNAC24	PH02Gene07883.t1	scaffold_14	297	32.71	8.74	Nucleus.	
PheNAC25	PH02Gene07908.t1	scaffold_14	393	42.76	9.13	Nucleus.	
PheNAC26	PH02Gene09530.t1	scaffold_17	327	36.47	6.46	Nucleus.	
PheNAC27	PH02Gene09730.t1	scaffold_23	332	38.47	6.18	Nucleus.	
PheNAC28	PH02Gene10707.t1	scaffold_16	300	32.81	8.87	Nucleus.	
PheNAC29	PH02Gene10725.t1	scaffold_16	408	44.03	8.86	Nucleus.	
PheNAC30	PH02Gene10908.t1	scaffold_3	741	81.26	4.68	Nucleus.	yes
PheNAC31	PH02Gene12270.t1	scaffold_13	357	39.41	6.31	Nucleus.	
PheNAC32	PH02Gene12296.t1	scaffold_18	390	41.89	6.31	Nucleus.	
PheNAC33	PH02Gene12630.t1	scaffold_3	907	99.32	4.87	Nucleus.	
PheNAC34	PH02Gene12645.t1	scaffold_3	340	37.76	9.04	Nucleus.	
PheNAC35	PH02Gene12819.t1	scaffold_19	750	82.73	6.24	Nucleus.	
PheNAC36	PH02Gene13021.t1	scaffold_6	375	40.95	6.56	Nucleus.	
PheNAC37	PH02Gene13454.t1	scaffold_21	344	38.75	6.41	Nucleus.	
PheNAC38	PH02Gene13734.t1	scaffold_12	318	34.90	9.18	Nucleus.	
PheNAC39	PH02Gene14487.t1	scaffold_8	372	40.61	6.56	Nucleus.	
PheNAC40	PH02Gene14824.t1	scaffold_24	336	36.86	6.65	Nucleus.	
PheNAC41	PH02Gene15458.t1	scaffold_22	322	35.56	6.83	Nucleus.	
PheNAC42	PH02Gene15515.t2	scaffold_9	307	33.71	8.82	Nucleus.	
PheNAC43	PH02Gene15869.t1	scaffold_6	349	37.27	8.79	Nucleus.	
PheNAC44	PH02Gene15919.t1	scaffold_21	358	38.86	8.84	Nucleus.	
PheNAC45	PH02Gene15922.t1	scaffold_21	354	39.04	5.81	Nucleus.	

PheNAC46	PH02Gene16115.t1	scaffold_22	364	40.32	9.34	Nucleus.	
PheNAC47	PH02Gene16123.t1	scaffold_22	374	40.33	6.34	Nucleus.	
PheNAC48	PH02Gene16242.t1	scaffold_13	141	15.82	9.68	Nucleus.	
PheNAC49	PH02Gene16245.t1	scaffold_13	317	34.68	6.14	Nucleus.	
PheNAC50	PH02Gene16285.t1	scaffold_17	650	71.31	4.53	Nucleus.	yes
PheNAC51	PH02Gene16580.t1	scaffold_13	287	31.93	9	Nucleus.	
PheNAC52	PH02Gene17008.t1	scaffold_4	301	34.26	5.93	Nucleus.	
PheNAC53	PH02Gene17549.t1	scaffold_2	368	41.18	7	Nucleus.	
PheNAC54	PH02Gene17994.t1	scaffold_4	342	37.34	6.86	Nucleus.	
PheNAC55	PH02Gene17999.t1	scaffold_4	340	37.66	5.64	Nucleus.	
PheNAC56	PH02Gene18494.t1	scaffold_6	524	58.17	5.37	Nucleus.	yes
PheNAC57	PH02Gene18734.t1	scaffold_8	353	37.75	7.71	Nucleus.	
PheNAC58	PH02Gene18901.t1	scaffold_10	315	35.50	6.53	Nucleus.	
PheNAC59	PH02Gene19087.t1	scaffold_5	327	36.08	6.21	Nucleus.	
PheNAC60	PH02Gene19279.t1	scaffold_15	353	38.94	5.83	Nucleus.	
PheNAC61	PH02Gene19280.t1	scaffold_15	360	38.91	8.5	Nucleus.	
PheNAC62	PH02Gene19527.t1	scaffold_13	388	41.67	8.2	Nucleus.	
PheNAC63	PH02Gene19528.t1	scaffold_13	366	40.86	5.82	Nucleus.	
PheNAC64	PH02Gene19631.t1	scaffold_14	292	32.68	6.1	Nucleus.	
PheNAC65	PH02Gene19632.t1	scaffold_14	344	37.55	7.08	Nucleus.	
PheNAC66	PH02Gene20080.t3	scaffold_14	349	40.29	6.62	Nucleus.	
PheNAC67	PH02Gene20102.t3	scaffold_14	650	71.41	5.2	Nucleus.	yes
PheNAC68	PH02Gene20630.t1	scaffold_15	312	34.10	8.64	Nucleus.	
PheNAC69	PH02Gene20719.t1	scaffold_13	325	35.76	5.66	Nucleus.	
PheNAC70	PH02Gene20743.t1	scaffold_2	321	35.17	6.32	Nucleus.	
PheNAC71	PH02Gene20782.t1	scaffold_17	206	22.83	6.29	Nucleus.	
PheNAC72	PH02Gene21429.t1	scaffold_6	341	38.44	6.7	Nucleus.	
PheNAC73	PH02Gene21591.t1	scaffold_15	661	73.65	5.96	Nucleus.	
PheNAC74	PH02Gene21727.t1	scaffold_12	260	28.08	9.79	Nucleus.	
PheNAC75	PH02Gene21976.t1	scaffold_3	355	38.70	6.92	Nucleus.	
PheNAC76	PH02Gene21998.t1	scaffold_24	279	30.62	7.78	Nucleus.	
PheNAC77	PH02Gene22100.t1	scaffold_11	360	39.97	8.59	Nucleus.	
PheNAC78	PH02Gene22459.t1	scaffold_16	321	34.70	5.61	Nucleus.	
PheNAC79	PH02Gene22560.t1	scaffold_13	341	38.15	5.11	Nucleus.	
PheNAC80	PH02Gene22723.t1	scaffold_15	308	33.87	5.76	Nucleus.	
PheNAC81	PH02Gene22867.t1	scaffold_16	738	80.39	5.99	Nucleus.	yes
PheNAC82	PH02Gene23384.t1	scaffold_15	333	37.60	7.62	Nucleus.	
PheNAC83	PH02Gene23860.t1	scaffold_17	241	27.41	8.85	Nucleus.	
PheNAC84	PH02Gene25003.t1	scaffold_17	356	38.89	6.64	Nucleus.	
PheNAC85	PH02Gene25075.t1	scaffold_4	278	31.23	5.5	Nucleus.	
PheNAC86	PH02Gene25195.t1	scaffold_13	324	35.77	7.1	Nucleus.	
PheNAC87	PH02Gene25626.t1	scaffold_17	316	35.38	5.89	Nucleus.	
PheNAC88	PH02Gene26002.t1	scaffold_13	264	29.04	8.44	Nucleus.	
PheNAC89	PH02Gene26525.t1	scaffold_24	202	22.51	5.54	Nucleus.	
PheNAC90	PH02Gene26991.t1	scaffold_3	275	30.54	5.99	Nucleus.	
PheNAC91	PH02Gene27508.t1	scaffold_13	355	40.21	8.1	Nucleus.	
PheNAC92	PH02Gene27577.t1	scaffold_16	334	36.75	7.68	Nucleus.	
PheNAC93	PH02Gene27578.t1	scaffold_16	334	36.75	7.68	Nucleus.	

PheNAC94	PH02Gene29132.t1	scaffold_7	456	50.43	4.66	Nucleus.	
PheNAC95	PH02Gene29164.t1	scaffold_15	316	35.52	5.69	Nucleus.	
PheNAC96	PH02Gene29456.t1	scaffold_8	313	34.57	7.1	Nucleus.	
PheNAC97	PH02Gene30294.t1	scaffold_3	244	27.58	5.14	Nucleus.	
PheNAC98	PH02Gene30295.t1	scaffold_3	217	25.27	8.14	Nucleus.	
PheNAC99	PH02Gene31596.t1	scaffold_6	309	33.92	7.72	Chloroplast. Nucleus.	
PheNAC100	PH02Gene31803.t1	scaffold_8	330	38.16	6.25	Nucleus.	
PheNAC101	PH02Gene32073.t1	scaffold_22	291	32.68	5.73	Nucleus.	
PheNAC102	PH02Gene32306.t1	scaffold_6	293	33.10	7.63	Nucleus.	
PheNAC103	PH02Gene33943.t1	scaffold_5	354	39.14	5.7	Nucleus.	
PheNAC104	PH02Gene34225.t1	scaffold_23	287	32.79	5.89	Nucleus.	
PheNAC105	PH02Gene34266.t1	scaffold_15	340	37.79	8.37	Nucleus.	
PheNAC106	PH02Gene34392.t1	scaffold_7	279	31.59	6.66	Nucleus.	
PheNAC107	PH02Gene34580.t1	scaffold_15	351	39.03	5.13	Nucleus.	
PheNAC108	PH02Gene34765.t1	scaffold_21	296	33.32	7.72	Nucleus.	
PheNAC109	PH02Gene36066.t1	scaffold_24	284	32.30	6.07	Nucleus.	
PheNAC110	PH02Gene36805.t1	scaffold_13	653	71.09	4.58	Nucleus.	yes
PheNAC111	PH02Gene37023.t1	scaffold_18	242	27.28	5.12	Nucleus.	
PheNAC112	PH02Gene37024.t1	scaffold_18	217	25.20	8.19	Nucleus.	
PheNAC113	PH02Gene37038.t1	scaffold_4	313	35.09	7.8	Nucleus.	
PheNAC114	PH02Gene37092.t1	scaffold_7	355	38.34	8.46	Nucleus.	
PheNAC115	PH02Gene37598.t1	scaffold_18	368	40.36	8.54	Nucleus.	
PheNAC116	PH02Gene37658.t1	scaffold_22	650	70.79	4.69	Nucleus.	yes
PheNAC117	PH02Gene38120.t2	scaffold_20	300	33.72	8.48	Nucleus.	
PheNAC118	PH02Gene39891.t1	scaffold_23	406	44.83	4.71	Nucleus.	
PheNAC119	PH02Gene40137.t1	scaffold_7	308	33.97	8.79	Nucleus.	
PheNAC120	PH02Gene40590.t1	scaffold_9	395	42.49	6.46	Nucleus.	
PheNAC121	PH02Gene40703.t1	scaffold_11	263	28.47	9.89	Nucleus.	
PheNAC122	PH02Gene41662.t1	scaffold_24	270	30.59	5.56	Nucleus.	
PheNAC123	PH02Gene42190.t1	scaffold_8	343	38.70	6.67	Nucleus.	
PheNAC124	PH02Gene42613.t1	scaffold_3	354	40.27	6.52	Nucleus.	
PheNAC125	PH02Gene43417.t1	scaffold_8	292	32.90	6.96	Nucleus.	
PheNAC126	PH02Gene43897.t1	scaffold_13	285	30.84	9.41	Nucleus.	
PheNAC127	PH02Gene44182.t1	scaffold_19	333	36.65	5.94	Nucleus.	
PheNAC128	PH02Gene44575.t1	scaffold_11	269	29.61	8.71	Nucleus.	
PheNAC129	PH02Gene44613.t1	scaffold_23	265	29.78	5.81	Nucleus.	
PheNAC130	PH02Gene44802.t1	scaffold_23	287	31.80	9.12	Nucleus.	
PheNAC131	PH02Gene45508.t1	scaffold_12	281	31.35	6.26	Nucleus.	
PheNAC132	PH02Gene45862.t1	scaffold_7	267	29.51	6.02	Nucleus.	
PheNAC133	PH02Gene46177.t1	scaffold_23	340	37.23	6.67	Nucleus.	
PheNAC134	PH02Gene46324.t1	scaffold_24	407	44.72	4.64	Nucleus.	
PheNAC135	PH02Gene47270.t1	scaffold_24	345	37.43	6.89	Nucleus.	
PheNAC136	PH02Gene48791.t1	scaffold_21	312	34.05	8.1	Chloroplast. Nucleus.	
PheNAC137	PH02Gene48835.t1	scaffold_21	165	18.84	9.86	Nucleus.	
PheNAC138	PH02Gene49501.t1	scaffold_22	334	37.31	5.96	Nucleus.	
PheNAC139	PH02Gene49650.t1	scaffold_10	307	34.79	6.03	Nucleus.	

PheNAC140	PH02Gene50550.t1	scaffold_21	358	38.93	9.16	Chloroplast. Nucleus.	yes
PheNAC141	PH02Gene31067.t1	scaffold_2	242	27.30	8.35	Nucleus.	
PheNAC142	PH02Gene25726.t1	scaffold_9	341	37.37	4.91	Nucleus.	
PheNAC143	PH02Gene01369.t1	scaffold_7	308	33.73	8.98	Nucleus.	
PheNAC144	PH02Gene12840.t1	scaffold_14	348	37.24	4.47	Nucleus.	
PheNAC145	PH02Gene42033.t1	scaffold_6	356	39.11	9.54	Nucleus.	
PheNAC146	PH02Gene48994.t1	scaffold_7	386	41.79	8.36	Nucleus.	yes
PheNAC147	PH02Gene09848.t1	scaffold_12	385	43.47	5.56	Nucleus.	
PheNAC148	PH02Gene00602.t1	scaffold_24	74	8.22	5.56	Nucleus.	
PheNAC149	PH02Gene13049.t1	scaffold_10	184	20.01	9.12	Nucleus.	
PheNAC150	PH02Gene50618.t1	scaffold_16	336	35.64	4.45	Nucleus.	
PheNAC151	PH02Gene19563.t1	scaffold_13	353	37.90	4.45	Nucleus.	
PheNAC152	PH02Gene05393.t1	scaffold_9	267	29.82	5.6	Nucleus.	
PheNAC153	PH02Gene10506.t1	scaffold_2	421	45.37	5.38	Nucleus.	
PheNAC154	PH02Gene08340.t1	scaffold_15	855	93.84	8.13	Golgi apparatus. Nucleus.	yes
PheNAC155	PH02Gene22362.t1	scaffold_13	211	23.37	8.8	Nucleus.	
PheNAC156	PH02Gene26426.t1	scaffold_16	503	54.58	6.55	Nucleus.	
PheNAC157	PH02Gene08339.t1	scaffold_15	446	49.19	5.43	Nucleus.	yes
PheNAC158	PH02Gene28157.t1	scaffold_8	399	44.67	5.42	Nucleus.	
PheNAC159	PH02Gene20473.t1	scaffold_16	126	14.61	6.41	Nucleus.	
PheNAC160	PH02Gene33256.t1	scaffold_23	561	61.05	7.59	Nucleus.	
PheNAC161	PH02Gene37212.t1	scaffold_3	345	38.32	9.24	Nucleus.	
PheNAC162	PH02Gene33764.t1	scaffold_3	242	28.36	10.56	Nucleus.	
PheNAC163	PH02Gene14934.t1	scaffold_16	199	22.65	6.84	Chloroplast.	
PheNAC164	PH02Gene36861.t1	scaffold_14	127	14.17	9.77	Chloroplast. Nucleus.	
PheNAC165	PH02Gene15473.t1	scaffold_16	248	27.19	5.56	Nucleus.	
PheNAC166	PH02Gene11440.t1	scaffold_2	236	25.97	5.97	Nucleus.	
PheNAC167	PH02Gene43360.t1	scaffold_16	319	35.09	5.89	Nucleus.	
PheNAC168	PH02Gene07864.t1	scaffold_14	458	51.14	4.61	Nucleus.	
PheNAC169	PH02Gene43882.t1	scaffold_8	90	10.60	9.39	Nucleus.	
PheNAC170	PH02Gene00727.t1	scaffold_16	381	40.61	4.47	Nucleus.	
PheNAC171	PH02Gene49020.t1	scaffold_24	143	15.43	5.5	Nucleus.	
PheNAC172	PH02Gene48445.t1	scaffold_1069	427	46.50	4.48	Nucleus.	
PheNAC173	PH02Gene19562.t1	scaffold_13	406	43.82	4.76	Nucleus.	
PheNAC174	PH02Gene36249.t1	scaffold_18	249	27.05	9.69	Nucleus.	
PheNAC175	PH02Gene15532.t1	scaffold_9	469	51.80	4.64	Nucleus.	
PheNAC176	PH02Gene18832.t1	scaffold_13	317	34.50	5.51	Nucleus.	
PheNAC177	PH02Gene27575.t3	scaffold_16	335	37.87	7.06	Nucleus.	
PheNAC178	PH02Gene18630.t1	scaffold_11	258	27.78	4.65	Nucleus.	
PheNAC179	PH02Gene03284.t1	scaffold_24	408	45.22	5.7	Nucleus.	
PheNAC180	PH02Gene19565.t1	scaffold_13	96	10.82	5.01	Nucleus.	
PheNAC181	PH02Gene04264.t1	scaffold_9	207	23.01	9.61	Nucleus.	
PheNAC182	PH02Gene45298.t1	scaffold_16	113	12.72	9.35	Nucleus.	
PheNAC183	PH02Gene42679.t1	scaffold_4	381	40.50	9.16	Nucleus.	
PheNAC184	PH02Gene06629.t1	scaffold_23	392	44.18	8.23	Nucleus.	

PheNAC185	PH02Gene11762.t1	scaffold_15	188	21.11	7.01	Nucleus.	
PheNAC186	PH02Gene05802.t1	scaffold_16	240	25.85	9.02	Nucleus.	
PheNAC187	PH02Gene47049.t1	scaffold_15	332	36.03	8.76	Nucleus.	
PheNAC188	PH02Gene20724.t1	scaffold_2	395	43.10	5.36	Nucleus.	
PheNAC189	PH02Gene08429.t1	scaffold_13	393	43.88	4.85	Nucleus.	
PheNAC190	PH02Gene51474.t1	scaffold_11189	136	15.34	8.63	Nucleus.	
PheNAC191	PH02Gene32948.t1	scaffold_8	456	49.69	7.81	Nucleus.	
PheNAC192	PH02Gene19564.t1	scaffold_13	353	37.88	4.45	Nucleus.	
PheNAC193	PH02Gene06293.t2	scaffold_14	423	47.77	7	Nucleus.	
PheNAC194	PH02Gene25869.t1	scaffold_9	317	33.90	4.57	Nucleus.	
PheNAC195	PH02Gene18668.t1	scaffold_11	372	40.60	9.02	Nucleus.	
PheNAC196	PH02Gene33138.t1	scaffold_14	256	27.83	5.56	Nucleus.	
PheNAC197	PH02Gene19561.t1	scaffold_13	353	37.85	4.45	Nucleus.	
PheNAC198	PH02Gene08630.t2	scaffold_13	255	27.87	9.93	Nucleus.	
PheNAC199	PH02Gene37378.t1	scaffold_5	493	55.03	5.29	Nucleus.	
PheNAC200	PH02Gene03749.t1	scaffold_14	513	58.32	9.16	Chloroplast. Nucleus.	
PheNAC201	PH02Gene14946.t2	scaffold_16	427	48.03	6.88	Nucleus.	
PheNAC202	PH02Gene10695.t1	scaffold_16	450	49.67	4.62	Nucleus.	yes
PheNAC203	PH02Gene50963.t1	scaffold_13393	152	15.92	4.82	Chloroplast. Nucleus.	
PheNAC204	PH02Gene37449.t1	scaffold_7	495	52.84	9.08	Nucleus.	
PheNAC205	PH02Gene04263.t1	scaffold_9	207	23.00	9.51	Nucleus.	
PheNAC206	PH02Gene41502.t1	scaffold_16	317	34.71	9.04	Nucleus.	
PheNAC207	PH02Gene00726.t1	scaffold_16	377	40.61	4.88	Nucleus.	
PheNAC208	PH02Gene22932.t2	scaffold_14	319	34.73	8.77	Nucleus.	
PheNAC209	PH02Gene50586.t1	scaffold_4341	353	37.83	4.46	Nucleus.	
PheNAC210	PH02Gene09879.t1	scaffold_12	418	45.75	4.41	Nucleus.	
PheNAC211	PH02Gene48447.t1	scaffold_2	434	47.36	4.42	Nucleus.	
PheNAC212	PH02Gene19621.t1	scaffold_3	250	27.26	9.89	Nucleus.	
PheNAC213	PH02Gene50619.t1	scaffold_16	309	32.51	4.87	Nucleus.	
PheNAC214	PH02Gene40100.t1	scaffold_9	427	47.77	9.35	Nucleus.	
PheNAC215	PH02Gene02567.t2	scaffold_18	579	63.63	4.69	Nucleus.	yes
PheNAC216	PH02Gene36515.t1	scaffold_5	233	25.48	6.5	Nucleus.	
PheNAC217	PH02Gene29743.t1	scaffold_14	139	15.27	6.51	Nucleus.	
PheNAC218	PH02Gene15879.t1	scaffold_16	94	10.62	7.88	Nucleus.	
PheNAC219	PH02Gene07274.t1	scaffold_14	250	26.86	8.98	Nucleus.	
PheNAC220	PH02Gene43555.t1	scaffold_12	243	26.58	6.05	Nucleus.	
PheNAC221	PH02Gene18568.t1	scaffold_3	629	71.81	8.56	Nucleus.	
PheNAC222	PH02Gene46116.t1	scaffold_22	251	27.41	10.25	Nucleus.	
PheNAC223	PH02Gene31682.t1	scaffold_8	139	16.26	10.15	Nucleus.	
PheNAC224	PH02Gene04042.t1	scaffold_23	372	41.84	5.42	Nucleus.	
PheNAC225	PH02Gene49642.t1	scaffold_13	211	23.11	7.76	Nucleus.	
PheNAC226	PH02Gene29174.t1	scaffold_15	355	38.52	5.68	Nucleus.	
PheNAC227	PH02Gene33776.t1	scaffold_3	246	27.35	8.46	Nucleus.	
PheNAC228	PH02Gene03449.t1	scaffold_12	293	31.82	8.89	Nucleus.	
PheNAC229	PH02Gene08342.t1	scaffold_15	344	37.93	5.52	Nucleus.	
PheNAC230	PH02Gene49686.t1	scaffold_6	229	25.30	8.24	Nucleus.	
PheNAC231	PH02Gene50122.t1	scaffold_24	436	49.12	9.52	Nucleus.	

PheNAC232	PH02Gene40661.t1	scaffold_15	120	13.99	10.37	Nucleus.	
PheNAC233	PH02Gene37201.t1	scaffold_3	155	17.12	6.51	Nucleus.	
PheNAC234	PH02Gene19823.t1	scaffold_15	146	16.01	5.69	Nucleus.	
PheNAC235	PH02Gene19730.t1	scaffold_12	202	22.48	8.89	Nucleus.	

Gene ID: refer to Bamboo Genome Datebase (BambooGDB, http://gigadb.org/dataset/100498); MW: molecular weight represents the predicted weights of PheNAC proteins; PI: represents the predicted isoelectric point of PheHsf proteins; MTFs: membrane-bound transcription factors.

Table S2. The list for primers.

Name	Forward Primer (5′ - 3′)	Reverse Primer (5′ - 3′)
PheNAC23 and *PheNAC23^{ES}* CDS sequence cloning		
CDS-PheNAC23	ATGAGCTTCTTGAGCAT	CTAGAAGGGATTCATCCAAGTAGAG
PheNAC23 and *PheNAC23^{ES}* overexpression construct		
OE-PheNAC23/23^{ES}	CGGGATCCATGTCCATGAGCTTCTTGAGCAT	CCCAAGCTTCTAGAAGGGATTCATCCAAGTAGAG
PheNAC23 and *PheNAC23^{ES}*-GFP fusion vector		
35S-EGFP-PheNAC23	GTTCATTTCATTTGGAGAGGACAGGATGTCCATGAGCTTCTTGAGCAT	TCGACTCTAGAGGATCCCCGGGTGAAGGGATTCATCCAAGTAGAG
35S-EGFP-PheNAC23^{ES}		TCGACTCTAGAGGATCCCCGGGTTGGCCAATGGTGCTGAG
GAL4 DBD fusion vector		
BD-PheNAC23/23^{ES}	GGCCGAATTCCCGGGGATGTCCATGAGCTTCTTGAGCAT	GCCGCTGCAGGTCGACGCTAGAAGGGATTCATCCAAGTAGAG
pGADT7-Rec2 fusion vector		
AD-PheNAC23/23^{ES}	GCAGAGTGGCCATTATGGCCCATGTCCATGAGCTTCTTGAGCAT	GCGGCCGACATGTTTTTTCCCCTAGAAGGGATTCATCCAAGTAGAG
BiFC fusion vector		
YN-PheNAC4 (pEarleyGate201-YN)	ACAAGTTTGTACAAAAAAATGTCCATGAGCTTCTTGAG	CACCACTTTGTACAAGAAGAAGGGATTCATCCAAGTAGA
PheNAC4ES-YC (pEarleyGate202-YC)	GGGGACAACTTTGTACAAAAAAGTTGGCATGTCCATGAGCTTCTTGAG	GGGGACAACTTTGTACAAGAAAGTTGGGCATGGCCAATGGTGCTGAGGT
qRT-PCR primers		
RT-PheNAC23	TTGGCCATGACACATCCACA	GGAGGTGTTGGTGGTGTTCA
RT- PheNAC23^{ES}	CCATGGGGAGGTAGCATGG	ATGGCCTGGGTAGTTCATGC
RT-PheTip41	AAAATCATTGTAGGCCATTGTCG	ACTAAATTAAGCCAGCGGGAGTG
RT-AtACT2	CGCTCTTTCTTTCCAAGCTC	AACAGCCCTGGGAGCATC
RT-AtFT	CTGGAACAACCTTTGGCAAT	TACACTGTTTGCCTGCCAAG
RT-AtFLC	TTAGTATCTCCGGCGACTTGAACCCAAACC	AGATTCTCAACAAGCTTCAACATGAGTTCG
RT-AtSOC	ACGAGAAGCTCTCTGAAAAG	GAACAAGGTAACCCAATGAAC
RT-AtAP2	ATATTTGGGACTGTGGGAAACAA	TGTCGGCGAAGTACGTGTACG

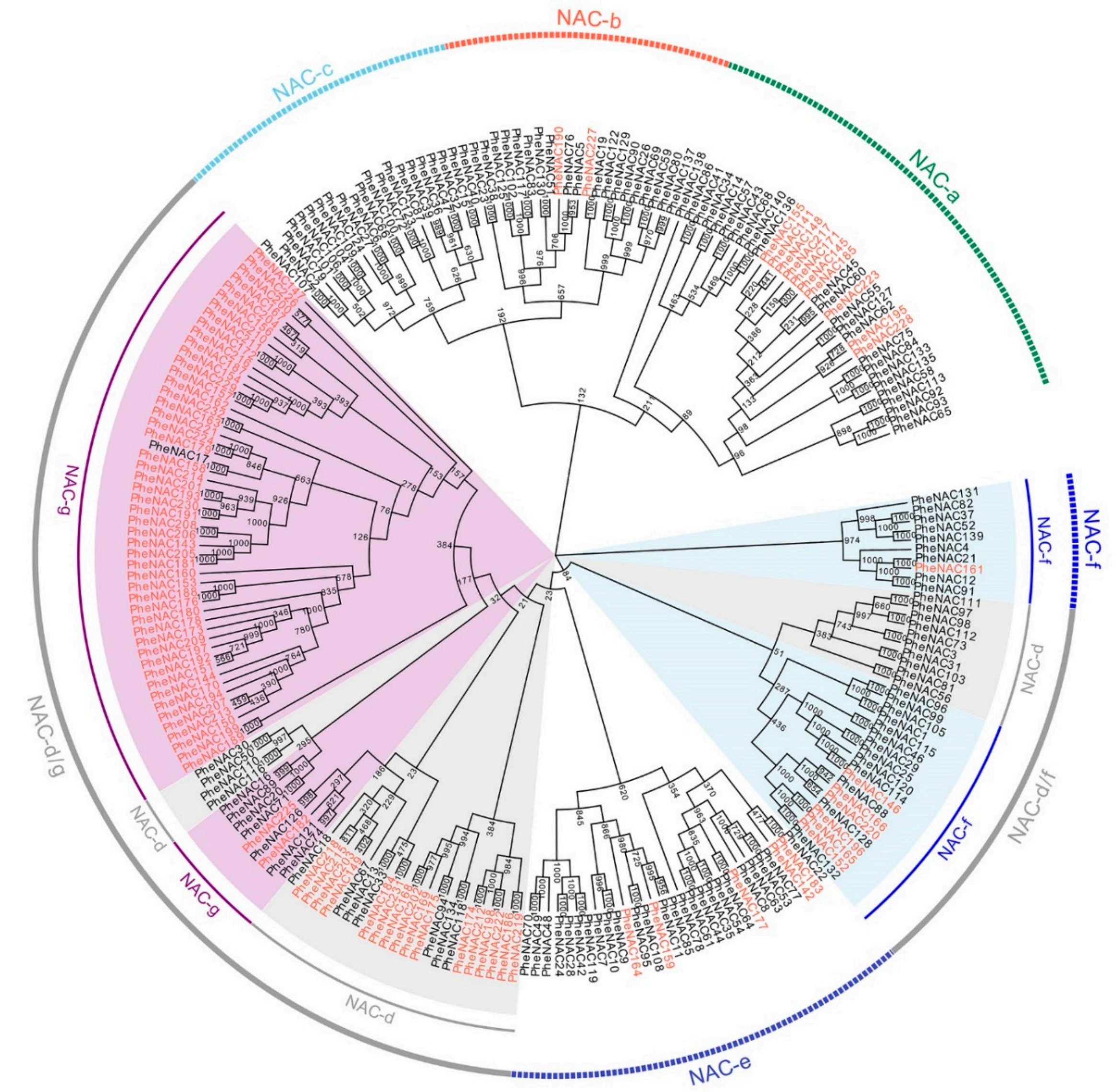

Figure S1. Phylogenetic relationships of the moso bamboo NAC genes.

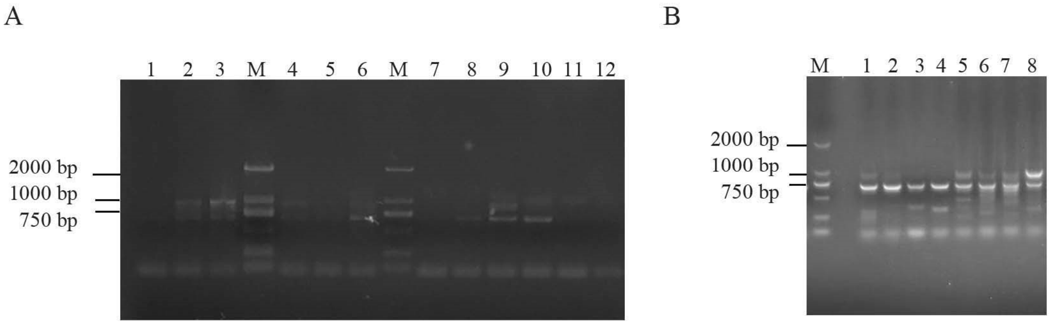

Figure S2. Cloning of the CDS sequence of the PheNAC23 gene. (A) lines 1-3: young, mature, and senescent stages leaves of three-year-old seedings of moso bamboo; lines 4-6: young, mature, and senescent stages leaves of six-month-old seedlings of moso bamboo, lines 7-12: floral bud formation stage, inflorescence growing stage, bloom stage, and embryo formation stage leaves of the flowering moso bamboo, M: DNA marker 2000; (B) leaves of two-month-old seedlings under PEG treatment, lines 1-9: 0 h, 1 h, 3 h, 6 h, 12 h, 24 h, 48 h, and 72 h, M: DNA marker 2000.
